# Restricted Genetic Diversity of HIV-1 Subtype C Envelope Glycoprotein from Perinatally Infected Zambian Infants

**DOI:** 10.1371/journal.pone.0009294

**Published:** 2010-02-18

**Authors:** Hong Zhang, Damien C. Tully, Federico G. Hoffmann, Jun He, Chipepo Kankasa, Charles Wood

**Affiliations:** 1 Nebraska Center for Virology, University of Nebraska, Lincoln, Nebraska, United States of America; 2 School of Biological Sciences, University of Nebraska, Lincoln, Nebraska, United States of America; 3 Department of Pediatrics, University Teaching Hospital, Lusaka, Zambia; University of California San Francisco, United States of America

## Abstract

**Background:**

Mother-to-child transmission of HIV-1 remains a significant problem in the resource-constrained settings where anti-retroviral therapy is still not widely available. Understanding the earliest events during HIV-1 transmission and characterizing the newly transmitted or founder virus is central to intervention efforts. In this study, we analyzed the viral *env* quasispecies of six mother-infant transmission pairs (MIPs) and characterized the genetic features of envelope glycoprotein that could influence HIV-1 subtype C perinatal transmission.

**Methodology and Findings:**

The V1-V5 region of *env* was amplified from 6 MIPs baseline samples and 334 DNA sequences in total were analyzed. A comparison of the viral population derived from the mother and infant revealed a severe genetic bottleneck occurring during perinatal transmission, which was characterized by low sequence diversity in the infant. Phylogenetic analysis indicates that most likely in all our infant subjects a single founder virus was responsible for establishing infection. Furthermore, the newly transmitted viruses from the infant had significantly fewer potential N-linked glycosylation sites in Env V1-V5 region and showed a propensity to encode shorter variable loops compared to the nontransmitted viruses. In addition, a similar intensity of selection was seen between mothers and infants with a higher rate of synonymous (dS) compared to nonsynonymous (dN) substitutions evident (dN/dS<1).

**Conclusions:**

Our results indicate that a strong genetic bottleneck occurs during perinatal transmission of HIV-1 subtype C. This is evident through population diversity and phylogenetic patterns where a single viral variant appears to be responsible for infection in the infants. As a result the newly transmitted viruses are less diverse and harbored significantly less glycosylated envelope. This suggests that viruses with the restricted glycosylation in envelope glycoprotein appeared to be preferentially transmitted during HIV-1 subtype C perinatal transmission. In addition, our findings also indicated that purifying selection appears to predominate in shaping the early intrahost evolution of HIV-1 subtype C envelope sequences.

## Introduction

In the global HIV-1 pandemic, mother-to-child transmission (MTCT) of HIV-1 remains a significant problem in the resource-constrained settings where the implementation of effective preventive and therapeutic strategies is still not widely available. In the absence of antiretroviral treatment, 30–45% of infants born to HIV-positive mothers become infected. Globally HIV-1 infected children account for 20% of all HIV-1 related deaths [1]. More than 90% of the infected children under the age of 15 are living in sub-Saharan Africa, where HIV-1 subtype C is the most prevalent and account for more than 60% of infections. HIV-1 Subtype C is now the most abundant and the most rapidly expanding subtype in the world. Given the high prevalence of HIV-1 subtype C infections, a better understanding of virus transmission is of significant importance.

Studies on perinatal transmission have mainly focused on HIV-1 subtype B infected individuals. Selective transmission of a few maternal variants has been frequently observed during MTCT [2], [3], [4], [5], although transmission of multiple or major maternal HIV-1 variants have also been shown in some studies [2], [3], [4], [6], [7], [8]. Growing evidence suggests that subtype C viruses may display characteristics that are distinct from subtype B and other subtypes, and that such differences may affect virus transmission and pathogenesis [9], [10]. A preliminary study from our group demonstrated that perinatal transmission of subtype C HIV-1 is consistent with most non-subtype C studies, selective transmission through the maternal-infant bottleneck was found to be dominant [11]. Although the basis for the maternal-infant transmission bottleneck remains poorly understood, it has been associated with specific viral selection [5], neutralization resistance [12], [13], [14], [15], [16] and enhanced replicative capacity [17] of the perinatally transmitted viruses. In addition, other factors such as selection for reduced glycosylation of gp120 could also be important [12], although this was not confirmed in other studies [13], [18]. Similar to perinatal transmission, a severe genetic bottleneck was also involved in heterosexual transmission with the transmitted virus containing a more compact and less glycosyloated Env for subtypes A and C [19], [20] but not for the majority of subtype B infections [19], [21], [22], [23]. In addition, the transmitted viruses through sexual transmission are either more susceptible [20] or resistant [22] to the donor neutralizing antibody. These studies highlight the complexity of transmission by different routes or subtypes and underscore the need to further explore the genetic and immunologic correlates of different subtypes or different routes of transmission.

In this study, we have further examined the perinatal transmission of HIV-1 subtype C of our Zambian mother-infant cohort. All the mother and infants are antiretroviral naïve, which have provided a setting to examine the origin and nature of transmitted viruses and determine the correlates of HIV-1 subtype C perinatal transmission without additional selective pressure. We have analyzed the viral *env* quasispecies of six MIPs and characterized the genetic features of *env* which could influence HIV-1 subtype C perinatal transmission. In particular, we have explored the selection pressures governing HIV-1 subtype C perinatal transmission. In agreement with the severe genetic bottleneck occurring during perinatal transmission among these MIPs, we find that the infant viruses are characterized by low sequence diversity and harbored significant evidence for restricted glycosylation in Env V1-V5 region. Our findings demonstrate the molecular characteristics of the virus which could be involved in HIV-1 subtype C MTCT and provide valuable insights into elucidating the dynamics of the MTCT.

## Results

### HIV-1 Infected Mother-Infant Pairs

All the infants were negative for HIV-1 isolation and PCR at birth, but were found to be HIV-1 positive either at 2 months (2617, 1449, 2669 and 2873) or 4 months (1084 and 1984) after birth. Thus, HIV-1 transmission most likely occurred either during delivery or through early breastfeeding. Because the amount of blood samples obtained from these infants was limited, priority was given to virus isolation in lieu of PCR when necessary (e.g., infant 1084, viral isolation was positive by 4 month but the first PCR was performed 6 months after birth). The first time point for positive PCR from each infant is indicated in Table 1. The primary isolates from these MIPs studied here exclusively used CCR5 as a co-receptor, exhibited macrophage-tropism, and did not infect T-cell lines or form syncytia *in vitro* ([24] and data not shown).

**Table 1:** Genetic variation between mother and infant in epidemiologically linked transmission pairs.

Patient ID	Subject	Months post-birth^a^	No of sequences	No of unique haplotypes	Diversity (%)^b^	dN/dS^c^	Median length (range) of V1-V5	Median PNGS (range) of V1-V5
1449	Mother Infant	*N/A* 2	37 26	37 21	6.13±2.72 0.48±0.15	0.77 0.71	337 (327–340) 330	23 (19–27) 21 (18–21)
2669	Mother Infant	*N/A* 2	32 30	32 29	3.16±0.90 0.71±0.29	0.83 0.54	340 (331–347) 339 (335–339)	26 (22–29) 25 (23–25)
2873	Mother Infant	*N/A* 2	31 29	30 26	5.25±2.25 0.60±0.28	0.81 0.91	352 (334–356) 356	26 (23–28) 28 (27–29)
2617	Mother Infant	*N/A* 2	27 27	26 27	0.77±0.23 0.63±0.32	0.59 0.52	338 (336–341) 336	24 (23–25) 23 (22–24)
1984	Mother Infant	*N/A* 4	30 25	29 25	2.53±1.4 1.11±0.43	0.88 0.89	339 (336–345) 343	24 (22–26) 21 (19–22)
1084	Mother Infant	*N/A* 6	41 25	27 25	5.35±3.1 1.38±0.47	0.98 0.62	333 (326–334) 319 (319–328)	23 (19–26) 21 (19–23)

a Maternal samples at delivery were defined as baseline and infant baseline samples were referred to the first postpartum HIV-1 PCR positive time point as indicated in months.

b Standard deviations for genetic diversity are indicated.

c dN/dS calculated after screening for recombination.

N/A means that this category is not applicable to maternal subjects.

### Phylogenetic Linkage of Transmission Pairs

**Figure 1:**
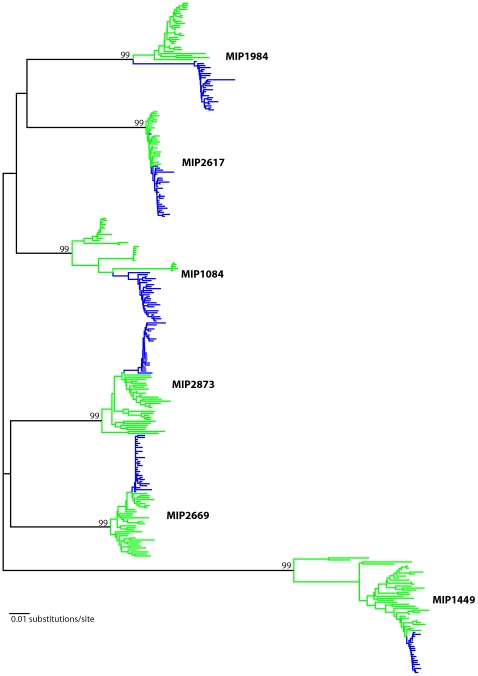
Neighbor-joining phylogenetic tree showing V1-V5 envelope gene sequences from all patients of the cohort with bootstrap values calculated from 1000 bootstrap replicates shown for each major branch. Each transmission pair is labeled with nucleotide sequences from mothers (green) and infants (blue). The tree was constructed using MEGA version 4.

The V1-V5 region of *env* gene was amplified from 6 MIPs and 334 DNA sequences in total were analyzed. When all sequences were included in a single phylogenetic analysis, sequences from each mother-infant pair formed a strong monophyletic group, indicating that maternal and infant sequences were epidemiologically linked and there was no evidence of cross-subject contamination (Figure 1). Subtyping analysis by comparing with reference sequences from the HIV Sequence Database indicated that all the MIPs were infected by subtype C, except for MIP 1449, which was a subtype A/C recombinant (data not shown).

**Figure 2:**
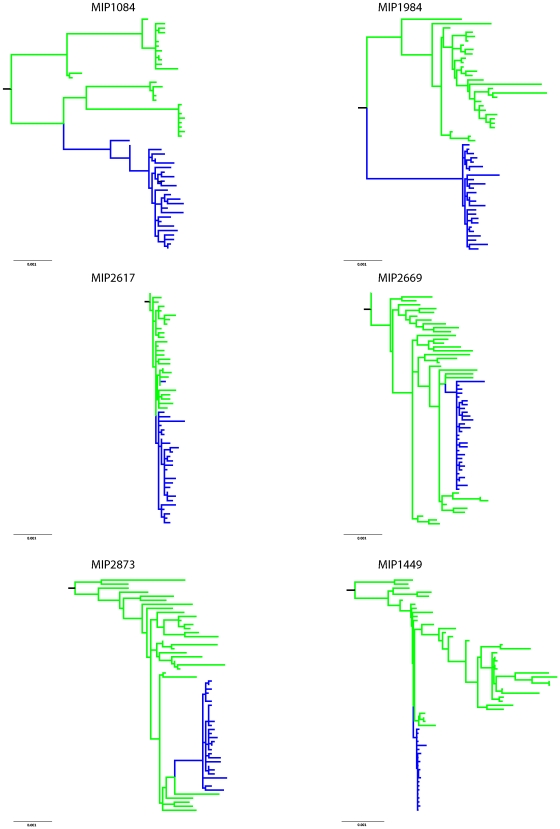
Rooted maximum-likelihood trees illustrating the epidemiologic linkage between sequences from mother (green) and infant (blue) subjects within each transmission pair. The nucleotide sequences of *env* V1-V5 region from each pair were aligned with two unrelated outgroup HIV-1 subtype C sequences obtained from the Los Alamos HIV Sequence Database. The branch containing unrelated outgroup sequences are not shown for space considerations. Only bootstrap support values greater than 75 are shown for the major nodes segregating mother and infant subjects.

To examine the evolutionary relationship of mother and infant sequences, maximum likelihood trees for each transmission pair were reconstructed. Phylogenetic analysis indicated clear epidemiological linkage between each mother and infant. This tree shape was consistent with the putative transmission events (Figure 2) where the infant population forms a distinct well-supported monophyletic cluster and is genetically less diverse than the maternal population. All but one infant's sequences are derived from a single branch of the maternal tree, suggesting a single variant being transmitted from the mother in 5 of the 6 transmission pairs. In the sixth, MIP2617, a single sequence branches differently on the tree from the bulk of infant sequences.

### Envelope Sequence Diversity of MIPs

The median number of *env* sequences analyzed per subject was 28.5 (range, 21–37) (Table 1). On average, the viral diversity in infants was 4.72 times lower than that in mothers (Table 1). The maximum within-patient *env* diversity for infant sequences was relatively low and ranged from 0.48% to 1.38%, with those infant samples amplified at 4 and 6 months demonstrating slightly elevated levels of variation as expected. In contrast, distinctly higher mean diversity was found in the maternal virus population and ranged from 0.77 to 6.13%. The diversities of the *env* sequences were statistically distinguishable between the mother and infant in each pair (*P* = 0.0087). This significant loss of mean genetic diversity is indicative of a severe genetic bottleneck during the transmission of HIV-1 subtype C from mothers to infants. Notably, for MIP 2617, the mean genetic diversity within the mother and infant was similar (0.77% versus 0.63%).

### Env Length of Maternal and Infant Variants

**Figure 3:**
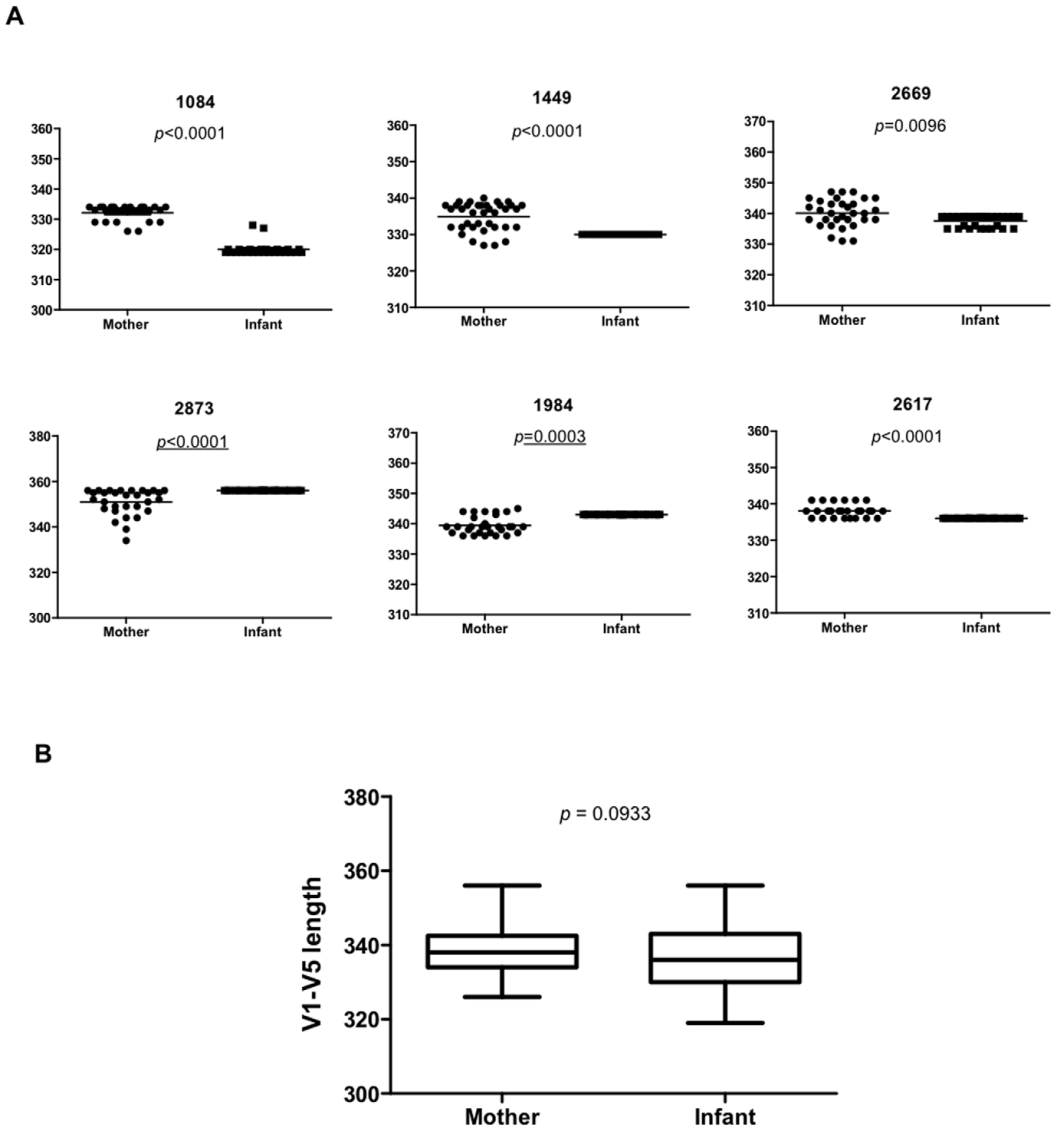
Comparison of Env V1-V5 length between mother and infant variants. (A) Comparison of Env V1-V5 length for each transmission pair. The *P* value for each comparison between the mother and infant are shown. The horizontal bars indicate the mean value for each individual. (B) Comparison of Env V1-V5 length of the aggregate sequences from mothers and infants. The *P* value for the comparison between the mother and infant is shown. The box and whiskers plot denotes the median, minimum and maximum values.

We further examined the genetic features of Env which could influence HIV-1 subtype C MTCT such as the length of Env. Comparison of Env V1-V5 length of maternal and infant variants indicated that in four of the six MIPs (MIP 1084, 1449, 2669 and 2617), the infant sequences had a significant shorter V1-V5 length than those of their mothers (Table 1 and Figure 3A, *P*≤0.0096). In contrast, for the remaining two pairs (MIP2873 and 1984), a significant increase in V1-V5 length in the infant sequences was observed (Table 1 and Figure 3A, *P*≤0.0003). When the data was considered in aggregate, there were no significant differences in Env V1-V5 length between the mothers (median, 338; range, 326 to 356; n = 181) and the infants (median, 336; range, 319 to 356; n = 153) although there was a trend toward the infant sequences having shorter Env V1-V5 length compared to the maternal sequences (Fig. 3B, *P* = 0.0933). Similar observations were obtained when the analysis was confined to V1-V2 and V1-V4 region with the majority of infant sequences harboring significantly shorter lengths compared to maternal sequences (Table 2). However, both the mother and infant sequences contained 35 amino acids in V3 region. Another interesting observation from the sequence data is the distinct uniformity in the length of the envelope glycoprotein in the infants (Tables 1 and 2). This uniformity is seen in the V1-V5 (Table 1), V1-V4 (Table 2), and even V1-V2 loops (Table 2) in all except two infants, with infant 2669 displaying a range of sequence lengths in the V1-V5 domain (Tables 1), while infant 1084 having a range of sequence lengths in V1-V5 and V1-V4 region (Table 1 and Table 2).

**Table 2:** Comparison of V1-V4 and V1-V2 domains between mother and infant in epidemiologically linked transmission pairs*.

Pairs	Amino acid length of V1V4			PNGS in V1V4			Amino acid length of V1V2			PNGS in V1V2		
	Mother (range)	Infant (range)	p value	Mother (range)	Infant (range)	P value	Mother (range)	Infant (range)	p value	Mother (range)	Infant (range)	P value
1449	280 (270–283)	275	<0.001	21 (18–24)	19 (18–19)	<0.001	73 (63–77)	67	0.003	7 (5–9)	5	<0.001
2669	287 (276–292)	284	0.002	22 (19–25)	21 (19–21)	<0.001	75 (61–77)	69	<0.001	8 (5–10)	7 (6–7)	<0.001
2873	296 (276–299)	298	<0.001	23 (20–26)	25 (24–26)	<0.001	74 (58–78)	74	0.01	7 (4–8)	7 (6–7)	0.072
2617	285 (283–288)	283	<0.001	20 (19–21)	20 (19–20)	0.748	74 (72–77)	72	<0.001	6 (5–7)	6 (5–6)	0.028
1984	287 (284–292)	289	0.003	21 (19–23)	19 (17–20)	<0.001	74 (71–81)	82	<0.001	7 (5–8)	6 (5–7)	<0.003
1084	277 (271–279)	267 (267–276)	<0.001	18 (13–20)	18 (16–20)	0.143	65 (60–68)	60	<0.001	4 (3–6)	5 (4–5)	0.938

* Median values for the length and the number of PNGS are shown.

### Restricted Glycosylation in Infant Variants

**Figure 4:**
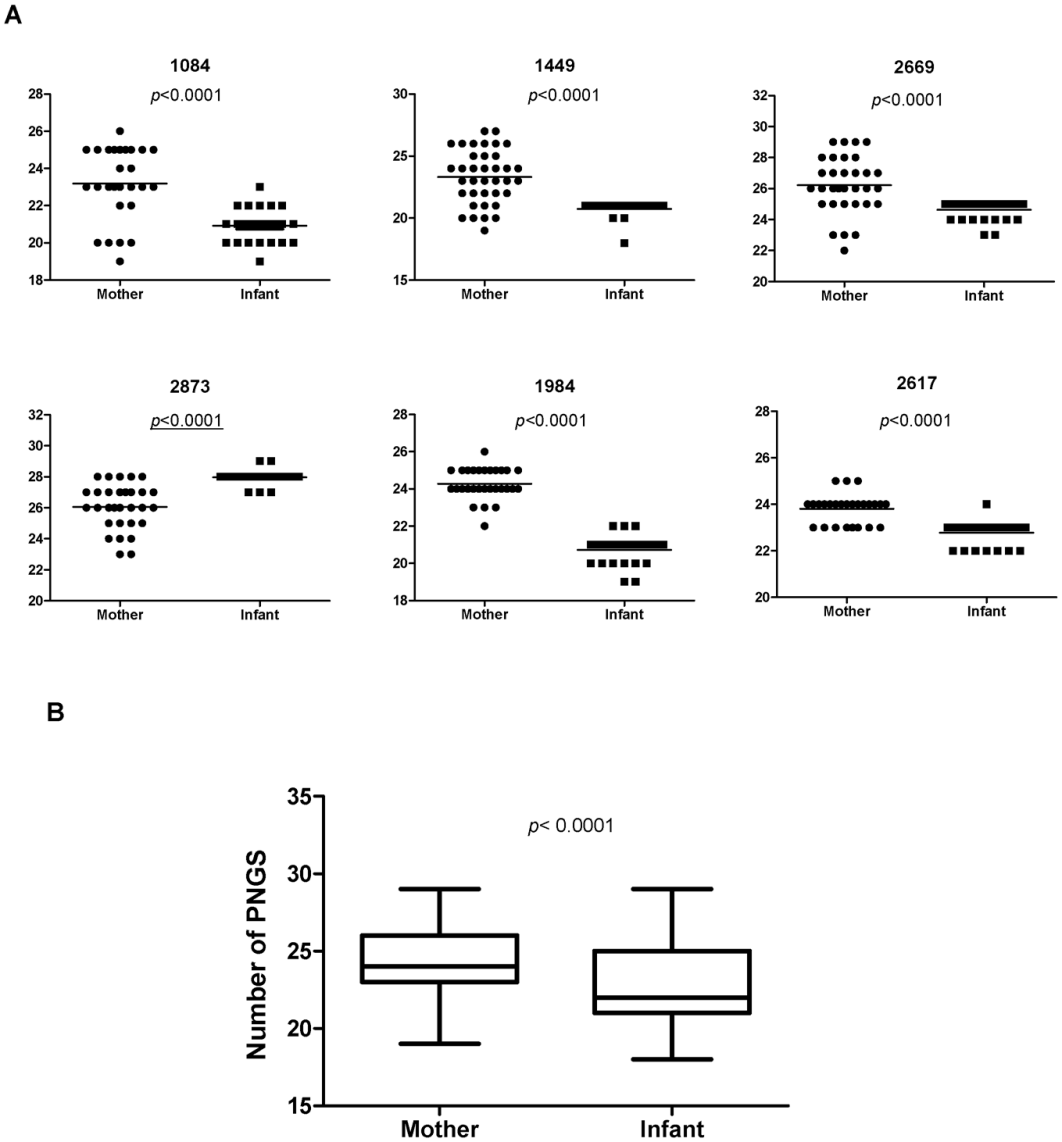
Comparison of PNGS in Env V1-V5 region between mother and infant variants. (A) Comparison of PNGS in Env V1-V5 region for each transmission pair. The *P* value for each comparison between the mother and infant are shown. The horizontal bars indicate the mean value for each individual. (B) Comparison of PNGS in Env V1-V5 region of the aggregate sequences from mothers and infants. The *P* value for the comparison between the mother and infant is shown. The box and whiskers plot denotes the median, minimum and maximum values.

The reduction in the variable loop size observed in the majority of the infant sequences led us to investigate the loss or addition of potential N-linked glycosylation sites (PNGS) in these regions. For five of the six pairs (MIP 1084, 1449, 2669, 1984 and 2617), the mothers had significantly greater numbers of PNGS in V1-V5 region than the infant sequences (Table 1 and Figure 4A, *P*<0.0001). In the remaining MIP2873, the median number of PNGS of infant sequences was greater than that of the mother (Table 1 and Figure 4A). Collectively, the observation of infant sequences (median, 22; range, 18 to 29) containing less than the number of PNGS in the mother (median, 24; range, 19 to 29) reached significance when the pairs were taken in aggregate for the V1-V5 domain (*P*<0.0001, Figure 4B). Restriction of these analyses to V1-V2 or V1-V4 of the envelope glycoprotein also indicated significant differences among MIPs, with most of the infant sequences showing fewer or equal number of N-glycans compared to the mothers' (Table 2). To specifically map the sites where the absence of PNGS in infant sequences occurred, we generated a consensus sequences from each subject and then aligned this sequence from each subject to the subtype B HIV-1 HXB2 sequence. The positions where PNGS are absent in sequences from infant but present in maternal sequence are summarized in Table 3. PNGS changes generally occurred in the V1, V2 or V4 region, as a result of insertion, deletion or mutations for most pairs (2669, 1449, 1984 and 1084). However, other conserved domains such as C2, C3 and C4 seemed to be susceptible to the loss or addition of PNGS as well.

**Table 3:** Positions of PNGS within the Env V1-V5 region that differed between paired mothers and infants^a^.

Subject ID	PNGS^b^	Cause^c^	Domain
MIP1449	133 185a 186 407 413	M I/D I/D M M	V1 V2 V2 V4 V4
MIP2669	133 136 355	I/D I/D M	V1 V1 C3
MIP2873	356 459a 463	M M M	C3 C4 V5
MIP2617	442	M	C4
MIP1984	144 160 190a 332 337 392 397 403 442 461	M M I/D M M I/D M M M I/D	V1 V2 V2 C3 C3 V4 V4 V4 C4 V5
MIP1084	136 289 396	M M I/D	V1 C2 V4

a Positions within the Env V1-V5 region where a PNGS was absent in infant variants but present in the paired mother variants.

b The PNGS positions are numbered according to the HXB2 amino acid sequence.

c Changes in the number of PNGS occurred as a result of either insertion/deletions (I/D) or mutation (M).

### Consistent Purifying Selection Pressure Across MIPs in *env*

To examine the selective pressures within individuals in each transmission pair, the dN/dS ratio was estimated for the V1-V5 region of *env*. The dN/dS range for the infants (0.52–0.91) was slightly lower than that of the mothers (0.59–0.98) but this difference was not significant (Table 1). In general, the infant and mother sequences of each transmission pair yielded similar magnitudes of dN/dS (<1) reflective of purifying selection operating across the V1-V5 region of *env* (Table 1). Although no positive selection was evident across the entire V1-V5 region of *env*, site-specific evidence for positive selection was found. In particular, the maternal sequences from 2669, 2617, 2873 and 1449 presented a limited number of codons (1 to 3) with site-specific positive selection. However, none of the infant variants showed evidence for positively selected codons perhaps owing to the limited time of virus diversification. Our screening for recombination employing GARD revealed the presence of recombination across all maternal sequences for each transmission pair, but only one significant recombination breakpoint was found in infant sequences belonging to 1084 and 1984 (data not shown).

## Discussion

Understanding the earliest events surrounding HIV-1 transmission and charactering the newly transmitted or founder virus is central to intervention efforts. In this study, we have analyzed the viral *env* quasispecies of six HIV-1 subtype C infected mother and infant pairs. Consistent with the majority of MTCT cases [2], [3], [4], [5], a transmission bottleneck was observed among all the MIPs and the transmitted viruses showed a consistent CCR5 dependence [24]. An essential feature of this bottleneck is that the infants harbored a less diverse HIV-1 population than their chronically infected mothers. In addition, the ML trees display a pattern indicating possible single variant transmission for 5 of the 6 pairs. However, the tree pattern for the remaining subject MIP2617 shows probable evidence for multiple variant transmissions. Although the short branch lengths and lack of variation segregating the maternal and infant population may suggest that this pattern is an artifact of the reconstruction process. To further substantiate this claim the clustering of this single infant sequence with the maternal sequences was not found to be as a result of contamination or even a labeling error during processing.

Multiple mechanisms could account for the observed selective transmission or outgrowth of detected variants during perinatal transmission. First, all the infants analyzed here were both HIV-1 PCR and viral isolation negative at birth, suggesting that they were infected either intrapartum or postpartum. Therefore, it is possible that the relative low viral load during delivery or in the breastmilk [25] of the chronically infected mothers may contribute to the transmission of the limited variants.

Second, MTCT occurs in the presence of passively acquired maternal antibody before birth. Therefore, maternal neutralizing antibody could impose a selective pressure on the transmitted virus. Our neutralization assay with these MIPs appeared to support this notion by showing that most of the newly transmitted viruses were more resistant to maternal neutralizing antibody compared to the maternal variants [16]. Similar results were also observed in subtype A or B infections [12], [13], [14], [15], suggesting both preventive and selective effects of maternal neutralizing antibody in perinatal transmission.

In addition, the bottleneck transmission may associate with the enhanced replicative fitness of the perinatally transmitted viruses. We previously demonstrated that in the MIPs analyzed here, the newly transmitted viruses were selected to have higher *ex vivo* fitness, as imparted by their Env V1-V5 region, than viruses from their chronically infected mother[17]. Therefore, the higher fitness may enable the transmitted virus to establish efficient replication in the new host and withstand the selection pressures imposed by mucosal barriers and neutralizing antibodies during the transmission.

Our results also suggest that the restricted glycosylation in Env V1-V5 region could be selected during transmission, with the transmitted variants having significantly fewer PNGS in envelope compared to those in the mother. This is consistent with and supports a previous study on subtype A perinatal transmission [12]. Thus, the reduced glycosylation in Env V1-V5 region may represent one major feature of the perinatally transmitted viruses. In addition, we found that there was a trend toward the infant sequences having shorter Env V1-V5 length compared to the maternal sequences. This difference may be attributed to founder events as a result of the transmission bottleneck with a compact Env glycoprotein descended from shorter sequences in the mother. Alternatively, they may have been descended from sequences that were originally longer but have been selected during transmission. The changes in the length and number of the N-glycan were mainly attributed to V1-V2 or V1-V4 region although glycan changes were also identified in C2, C3 or C4 regions. Structural studies have suggested that V1-V2 loop may play a role in occluding CD4 and a coreceptor binding site [26], [27], [28]. In addition, V4 region has been implicated in the Env conformation change and glycan packing [29]. Thus, decrease in the length or removal of glycan from these regions could potentially expose the binding site for CD4 or coreceptor, thereby influcing viral infectivity or susceptibility to neutralizing antibody.

Among our study cohort, infants 2617, 1449, 2669 and 2873 died within the first year after birth and are characterized as fast progressors. On the other hand, infants 1084 and 1984 were diagnosed as slow progressors. Phylogenetic analysis of the *env* sequences revealed that all six MIPs were epidemiologically linked and well discriminated. In addition, the restricted genetic diversity was observed in all infected infants, irrespective of the timing of transmission or disease progression. MIP2617 is also unique in that the genetic diversity of the maternal viral population was very low (0.77%). This could be due to the lack of effective neutralization response in the mother, since neutralizing antibody could have substantial effects on the evolution of Env [22].

Concordant with a previous study [30], we concluded that intrahost HIV-1 evolution is predominantly affected by purifying selection. In this regard, our observations of a small number of positively selected codons in maternal variants may arise from a complex combination of factors that perpetuates the “arms race” between host and virus. On the other hand, the scarcity of positively selected sites in the infants may be due to the naïve state of the neonatal immune system with the effective cellular immune response to HIV-1 in infants being delayed [31], [32], [33], [34]. In addition, the lack of significant elevation in dN/dS between the mother and infants in this study is similar to previous reports studying heterosexual transmission between the source and recipient partners [20], [23] and those following pediatric transmission [35]. Our observation of recombination occurring in maternal isolates is not unexpected because diversity is likely to vary greatly depending on the stage and duration of infection. Furthermore, the observation of an increase in the proportion of detected recombination events in the infant variants 1084 and 1984 may be attributed to an increase in viral sequence diversity.

One of the limitations in this study is that single genome amplification was not performed on these samples since these samples were processed and amplified earlier [24]. Unfortunately, there was insufficient amount of materials needed to perform this technique. Nevertheless, in order to eliminate the confounding effects of traditional molecular cloning approaches such as *in vitro* PCR recombination, a phylogenetic analysis was performed. This phylogenetic analysis allows us to distinguish between PCR-induced recombination and actual *in vivo* recombination events. This simple analysis revealed that none of the clones was as a result of *in vitro* PCR recombination. Besides, our recent study also indicated that PCR mediated recombination did not obscure the phylogenetic analysis of *env* during the disease progression of a rhesus macaque infected with a CCR5- usage SHIV-C [36].

In summary, our findings demonstrated that the newly transmitted viruses from the infant are phylogenetically less diverse with a marked reduction in Env length and significantly fewer PNGS compared to the maternal viruses. The restricted genetic diversity observed in the infant is probably owing to the fact that a single HIV-1 variant from the maternal quasispecies initiated the infection. In addition to the transmission bottleneck, our study also revealed that the evolution of *env* gene in infants was dominated by purifying selection. Further dissecting the biological factors favoring transmission through the maternal-infant bottleneck, particularly characterizing newly transmitted or founder viruses, will be critical in understanding the molecular mechanism of MTCT and in guiding novel intervention approaches.

## Materials and Methods

### Patient Information and Sample Collection

Six mother-infant pairs (MIPs) labeled 2617, 1449, 1084, 2669, 2873 and 1984 from our Zambian mother-infant cohort [24] were characterized in this study. The mothers were known to be HIV-1 positive at the time of delivery and all subjects were asymptomatic. The infants were all breast-fed and drug naïve. Venous blood was collected from the mother before delivery and from the infant within 24 hours of delivery and at subsequent time points. Maternal samples at delivery were defined as baseline and infant baseline samples were referred to the first postpartum HIV-1 PCR positive time point. The baseline HIV-1 serological status of the mother and HIV-1 infection in infants was determined as previously described [37], [38]. This study was approved by the Zambian Ministry of Health, the Research and Ethics Committee of the University Teaching Hospital in Lusaka, Zambia and the Institutional Review Board of the University of Nebraska, Lincoln and was conducted in accordance with the principles of the Helsinki Declaration. All study participants or their parents provided written informed consent for the collection of samples and subsequent analysis.

### Cloning and Sequencing of *env*

For amplification of proviral HIV-1 *env* gene, genomic DNA was extracted from uncultured peripheral blood mononuclear cells (PBMC) using the Puregene kit (Gentra Systems) for all subjects except mother 1084. For mother 1084, HIV-1 *env* gene was amplified from placenta tissue since PBMC was not available. Nested PCR was used to amplify a 1100 bp fragment spanning the V1-V5 region of the *env* gene. In order to minimize the PCR bias, first round PCR products were generated in duplicates, combined and used as the template in a second-round PCR. Primers and PCR parameters used were described previously [24]. Amplified fragments were cloned into the pGEM-T Easy vector (Promega) and sequenced in both directions with dideoxy terminators (ABI BigDye Kit). A total of 25-37 clones were sequenced for each sample to obtain a representative measurement for the diversity of the viral genotypes.

### Phylogenetic Analysis

Nucleotide sequences were aligned with Clustal W version 1.4 [39] and manually refined using MacClade v4.05 software. A maximum likelihood (ML) tree was constructed for each transmission pair, which included two unrelated subtype C reference sequences from the Los Alamos HIV Sequence Database as outgroup sequences to root the trees. After gap-stripping, Modeltest [40] was used to identify the optimal evolutionary model as assessed by the hierarchical likelihood ratio test. PAUP* version 4.0b10 [41] or PhyML [42] was then used to calculate a ML tree for each pair incorporating the optimal model and its parameters from Modeltest in a heuristic search with a neighbor-joining start and SPR branch swapping. Viral diversity was estimated as the average pair-wise genetic distance between all sequences within a patient and was calculated using PAUP* version 4.0b10 with the corresponding evolutionary model as defined by Modeltest.

### Potential N-Linked Glycosylation Sites (PNGS)

Potential N-linked Glycosylation sites (PNGS) were predicted using N-GlycoSite from the Los Alamos National HIV database [43] (http://www.hiv.lanl.gov/content/sequence/GLYCOSITE/glycosite.html).

### Selection Analyses

For each transmission pair, selection within individuals was quantified using the ratio of nonsynonymous to synonymous substitutions (ω = dN/dS). Positive selection, neutral evolution and purifying selection is indicated by ω>1, ω = 1 and ω<1 respectively. To identify site-specific selection we used a single likelihood ancestral counting (SLAC), fixed effects likelihood (FEL) and random effects likelihood (REL) model implemented in the HyPhy (www.hyphy.org) software package [44] and Datamonkey web-interface [45]. In order to maintain confidence in the inference of codon sites under selection only sites corroborated by each method were taken as “true” positives. This was done to mitigate against false positives and negatives, as SLAC is a conservative test that may miss some selected sites while a REL approach may suffer from high rates of false positives detecting more sites [46].

Maximum likelihood methods of codon substitution may suffer from high rates of false positives when the sequences being analyzed have undergone recombination [47], [48]. Therefore, we first screened for recombination in all our data sets by identifying breakpoints and segment-specific phylogenies, using a genetic algorithm for recombination detection (GARD) [49], [50]. This method searches for breakpoints in the alignment, inferring phylogenies for each putative nonrecombinant fragment. In each case the nucleotide substitution matrix was optimized for each data set with Beta-Gamma rate variation and three rate classes as initial running parameters.

### Statistical Analysis

To compare the length and number of PNGS between mother and infant sequences, we used a non-parametric Mann-Whitney test. *P* values of less than or equal to 0.05 were considered significant and a trend was reported when 0.05<*P*≤0.1. All statistical tests were performed with GraphPad Prism software version 5.0 (Graph Pad Software Inc., San Diego, CA) and SPSS version 16 (SPSS, Chicago, IL).
