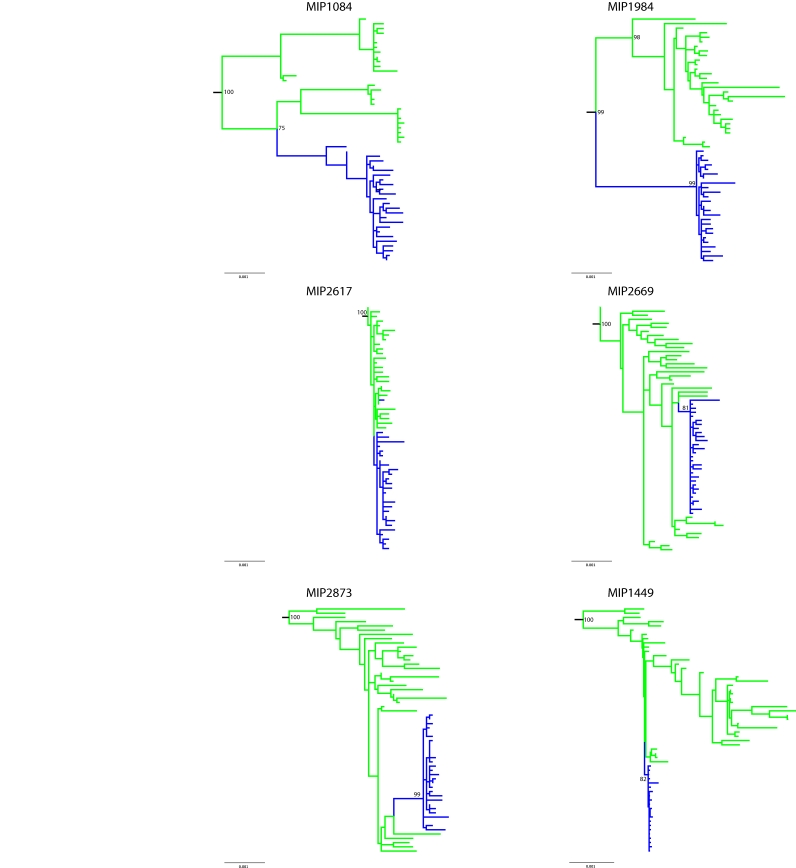# Correction: Restricted Genetic Diversity of HIV-1 Subtype C Envelope Glycoprotein from Perinatally Infected Zambian Infants

**DOI:** 10.1371/annotation/eadee58d-56c0-4399-b007-d80cc71e3f8a

**Published:** 2010-03-09

**Authors:** Hong Zhang, Damien C. Tully, Federico G. Hoffmann, Jun He, Chipepo Kankasa, Charles Wood

In Figure 2, the bootstrap support values indicated in the legend have been omitted in the figure. Please view the correct Figure 2 here: 

**Figure:**