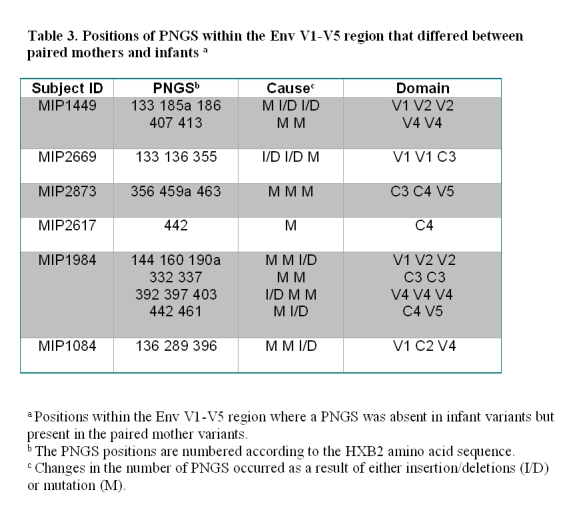# Correction: Restricted Genetic Diversity of HIV-1 Subtype C Envelope Glycoprotein from Perinatally Infected Zambian Infants

**DOI:** 10.1371/annotation/c2dd3548-93b7-480e-991a-93a728bca5fe

**Published:** 2010-03-09

**Authors:** Hong Zhang, Damien C. Tully, Federico G. Hoffmann, Jun He, Chipepo Kankasa, Charles Wood

In the fifth row of Table 3, the data in the second to fourth columns are misaligned. Please view the correct table here: 

**Figure:**